# Authors' reply

**Published:** 2010

**Authors:** Mihir Kothari, Suwarna Balankhe, Rinkle Gawade, Svetlana Toshnival

**Affiliations:** 1 Mahatme Eye Hospital and Eye Bank, 16 Central Excise Colony, Chhatrapati Square, Wardha Road, Nagpur, India; 2 Jyotirmay Eye Clinic and Pediatric Low Vision Center, 205, Ganatra Estate, Pokhran Rd No. 1, Khopat, Thane West – 400 601, India; 3 Aditya Jyot Eye Hospital, 153, Maj. Parmeshwaran Rd No 9, Wadala, Mumbai–400 031, Maharashtra, India

Dear Editor,

We are thankful to Gogate[1] for his interest in our article.[2] We agree that the results of a hospital-based study could differ from a population-based study due to a selection bias. This would be true for any kind of epidemiological survey for such a disease. Hence, in agreement with Gogate, we believe it would be interesting and desirable to repeat this study in the population.

A follow-up study after squint correction is also desirable. The readers can refer to a study by Archer *et al.*[3] They demonstrated that statistically significant improvements can be seen in the social, emotional, and functional measures of the children's health status after surgical realignment. This is mentioned in the discussion section of our manuscript.[2]

For a sample of 16 and 77, 68% and 61% respectively cannot be called as an obvious gender bias. Using a confidence interval (CI) approach, for 95% CI, true population proportion for 11/16 could range from 46-91.5% and that for 47/77 could range from 50-72% just by chance. Invoking a hypothesis of a gender bias and then providing an explanation for such a bias is dangerous and should be done in a study with a larger sample size. We have mentioned the *P* value for gender difference in our manuscript.[2]

As a matter of fact, we decided to give more weightage to maternal reporting to reduce the recall (report) bias in the study. In the past, during history-taking for patients with strabismus, we frequently encountered situations in which the mother would give more accurate history. Frequently, the mother had noticed the squint much before anyone else had noticed the squint. In most Indian families, mothers spend more time with school-going children than the fathers. Although this is not a rule, it is clear from the previous studies that the reliability of parental reporting in family studies is biased towards maternal reporting.[4]

The rural group included patients from the most under-served area of the society. They came from the tribal belt of Vidarbha region of Maharashtra. The surgery was done at absolutely no cost. The parents and children were provided with free food and free transport.

## References

[1] Gogate P (2010). Psycho-social and emotional impact of strabismus on Indian families. Indian J Ophthalmol.

[2] Kothari M, Balankhe S, Gawade R, Toshnival S (2009). Comparison of psychosocial and emotional consequences of childhood strabismus on the families from rural and urban India. Indian J Ophthalmol.

[3] Archer SM, Musch DC, Wren PA, Guire KE, Del Monte MA (2005). Social and emotional impact of strabismus surgery on quality of life in children. J AAPOS.

[4] Donoghue EC, Shakespeare RA (2008). The reliability of paediatric case-history milestones. Dev Med Child Neurol.

